# Supplementary material for: Assessment of treatment outcomes and associated factors among patients treated with clozapine at amanuel mental specialized hospital, Addis Ababa, Ethiopia
Source: PLoS One. 2025 Apr 16;20(4):e0321536. doi: 10.1371/journal.pone.0321536 (PMC12002802; doi:10.1371/journal.pone.0321536)
Supplement: Attachment — Submitted filename: Response to reviewers.docx [file pone.0321536.s003.docx]

**Response to reviewers**

We want to thank the reviewers for critically examining the manuscript and providing valuable comments. We are confident that the comments will greatly improve the quality of the manuscript. We have addressed the comments and improved the manuscript accordingly to the best of our capacity.

**Our responses:**

**Reviewer 1:**

***Methods:***

1. Sample size and duration: Due to limited number of patients who were on treatment with clozapine in the hospital, we decided to include all those eligible in the study. We are aware that this will be a limitation of the study and have acknowledged this fact in the discussion. The duration for the study was the time taken to include all eligible participants when they visited the hospital for follow-up. We have added statements in the manuscript addressing this issue for the purpose of clarity.
2. Using CGI without consistent use of PANSS and AIMS: Since this is a retrospective study compounded by cross-sectional design, the use of assessment tools such as PANSS and AIMS were not used consistently during treatment. However, thorough clinical assessments were made at the time of all progress and follow-up evaluations. Again, this is a limitation of the study and we acknowledged this in the limitations section as you suggested. We suggest experimental studies with controls to verify the findings of our study and included such suggestion in the conclusion part.
3. Variable selection: The variable selection was not arbitrary but based on factors described in the literature; additional factors which were considered to impact treatment outcome of clozapine from clinical experience were included. We did not find several studies which described factors associated with outcome of clozapine treatment in the literature.

***Results:***

1. Variable representation: the variables included in the study are all relevant to the study because the study explores not only outcome of clozapine treatment but also factors associated with good outcome. Several variables were included in order to identify the factors which had true effect on outcome of treatment from a relatively long list of factors. Therefore, we don’t believe including those variables would affect focus of the study. The clinical characteristics of participants listed in Table 2 included factors which were considered to affect treatment outcomes. Along with socio-demographic, stress and social support, and substance use characteristics, these clinical characteristics can describe the study participants. The table, however, shows the clinical characteristics most relevant to the study. The effect of substance use was analyzed to check for presence of associations with treatment outcomes. However, the study did not find any associations of substance use. Therefore, substance use was included for exploration and was explored but did not show any association. That was why it was not discussed further in the results and discussion.

***Discussion:***

1. Contextualization of findings: As you commented, we have found and included a previous study conducted in Ethiopia to contextualize our findings. We believe the inclusion of this study in the discussion section will greatly address the issue of contextualizing the findings of our study. We have also discussed how our study differs from this previous study. We have included this in the revised manuscript.

***Conclusion:***

1. ***Limitations and practical implications:*** We have addressed the small sample size in the limitations section, as well as elaborated it in the methods section. We have added the limitations due to the study design (retrospective) in the limitations section based on your comment. We believe readers would understand the limitaions from the elaborations in the methods section and we also emphasized them in the limitations section. But we believe our study doesn’t fail to offer practical implications for clinical practice as the findings of the study clearly show the benefits of clozapine. We have also added the importance of randomized clinical trials to verify the findings of our study given its limitations. We have recommended future well designed studies. Our study is just one study and further studies will provide further evidence for clinical and academic purposes. As for addressing the long-term implication of clozapine treatment, we leave it for future studies because it is not the objective of our study to show the long-term effects of treatment with clozapine.

**Reviewer 2:**

Based on your comments we have elaborated the ***methods*** section. We have addressed your comments as follows:

- We have modified the study design and included it in our revised manuscript as “… retrospective study design compounded by cross-sectional design” We have also specified which data were retrospective and which ones were cross-sectional in the relevant areas of the methods section.
- We have made amendments to the abstract section as well.
- The mean duration of hospitalization was between 32 and 35, but the guideline recommended minimum of one month. Patients were discharged after one month except they can take few days during discharge process.
- We have addressed how adherence was ensured after discharge by stating “… based on the guideline… patients with a trusted caregiver were initiated on clozapine… caregivers were given health education… caregivers supported patients to adhere to treatment after they were convinced clozapine was the only remaining hope for better outcome.”
- There was no access to measures of therapeutic levels of clozapine and we have included a statement in the methods section to describe this fact. We also mentioned it in the limitations section.
- The psychiatric diagnosis of all the patients with TD was schizophrenia and we have included a statement in the methods section to describe it.
- The pathway to clozapine treatment for TRS was at least one of the medications which failed was a second generation antipsychotic (risperidone or olanzapine) and we have included a statement in the revised manuscript to describe this.
