# Supplementary material for: Assessment of treatment outcomes and associated factors among patients treated with clozapine at amanuel mental specialized hospital, Addis Ababa, Ethiopia
Source: PLoS One. 2025 Apr 16;20(4):e0321536. doi: 10.1371/journal.pone.0321536 (PMC12002802; doi:10.1371/journal.pone.0321536)
Supplement: Attachment — Submitted filename: Review comments to the author.docx [file pone.0321536.s002.docx]

**Review comments to the author**

Thanks for adequately addressing all the comments.
