# Supplementary material for: Assessment of treatment outcomes and associated factors among patients treated with clozapine at amanuel mental specialized hospital, Addis Ababa, Ethiopia
Source: PLoS One. 2025 Apr 16;20(4):e0321536. doi: 10.1371/journal.pone.0321536 (PMC12002802; doi:10.1371/journal.pone.0321536)
Supplement: Attachment — Submitted filename: Response_to_reviewers_auresp_2.docx [file pone.0321536.s004.docx]

**Response to reviewers**

Dear reviewers, thank you for your constructive comments. I believe the comments have significantly improved the quality of the manuscript so far and will do so this time too. I have addressed the remaining comments as follows:

1. The reason why patients stayed in hospital for one month was clarified in the manuscript. The reason for one month admission was for initiation of clozapine. Once stabilized, the patients were discharged from hospital and continued their treatment as outpatient.
2. The average dose of clozapine was 296 mg and this was included in the results section, clinical characteristics subsection of the manuscript.
3. The nature of side effects identified was mild forms of sedation, hypersalivation and occasional urinary incontinence. A statement is added to results section, clinical characteristics subsection of manuscript.
4. No agranulocytosis was reported and all participants were continuing their treatment with monitoring. A statement is added to the results section, clinical characteristics subsection of manuscript.
5. There was no problem reported about compliance to blood count and ESR monitoring of the patients and this is addressed also in the manuscript.
6. The psychoactive substances used are already in the manuscript. The reported ones were khat and cigarettes.
7. We have gone through the manuscript for proof reading and we have made several editions to grammar and punctuation to the manuscript.

Finally, we hope this will satisfy the requirements based on your comments.

With regards
